# Noncommunicable Diseases: A Globalization of Disparity?

**DOI:** 10.1371/journal.pmed.1001859

**Published:** 2015-07-28

**Authors:** Peter J. Hotez, Larry Peiperl

**Affiliations:** 1 Department of Pediatrics and Molecular Virology and Microbiology, National School of Tropical Medicine, Baylor College of Medicine, Houston, Texas, United States of America; 2 Sabin Vaccine Institute and Texas Children’s Hospital Center for Vaccine Development, Houston, Texas, United States of America; 3 James A. Baker III Institute for Public Policy, Rice University, Houston, Texas, United States of America; 4 Department of Biology, Baylor University, Waco, Texas, United States of America; 5 Public Library of Science, San Francisco, California, United States of America

## Abstract

Peter Hotez and Larry Peiperl argue that the world’s poorest people may take on a disproportionate burden of noncommunicable diseases, even as their home countries gain in economic power.

A year ago, the editors of *PLOS Neglected Tropical Diseases* and *PLOS Medicine* launched the PLOS Blue Marble Health Collection, subtitled “the mismatch between national wealth and population health” [1]. The term “blue marble health” (which recalls the appearance of the earth from space) was coined as a differentiator from prior conceptualizations of global health that divided the world’s population according to national economic indices [2]. The basic tenet of blue marble health is that impoverished populations living amidst wealth bear a disproportionate burden of neglected diseases, irrespective of the overall economic strength of their home country. Such an approach is increasingly relevant as differential disease burdens between wealthier countries and regions (including North America, Europe, and Japan) and lower-income countries (including many in Africa, Asia, and Central and South America), evolve through a pronounced, but uneven, economic rise across the planet that leaves pockets of intense poverty in its wake.

The concept of blue marble health grew out of a paradoxical observation by one of us (PJH) that most of the world’s neglected tropical diseases (NTDs) are found in concentrated areas where poor people live in the wealthiest economies, especially the group of 20 (G20) countries together with Nigeria [2]. A follow-up study published this week in *PLOS Neglected Tropical Diseases*, based on newer data released by the World Health Organization (WHO) for the year 2013, finds that at least one-half or more of the world’s helminth infections and most of the dengue fever cases are also endemic to the G20 nations and Nigeria [3].

A recent analysis, based on data from WHO and the Joint United Nations Programme on HIV/AIDS (UNAIDS), indicates that lack of protection by national economic power extends beyond the NTDs to include the so-called “big three diseases”: HIV/AIDS, tuberculosis, and malaria; overall, roughly one-half of the prevalent cases of tuberculosis and malaria in 2013 were found in the G20 and Nigeria, as were almost one-half of the people living with HIV/AIDS [4].

The policy implications of blue marble health related to infections include emphasizing the G20’s role in mass treatment of their own indigenous populations for NTDs; prevention and appropriate screening for HIV, TB and malaria; and committing resources to conduct research and development (R&D) [5]. The need for such a policy is based partly on studies that point to disproportionately small contributions by the BRICS (i.e., Brazil, Russia, India, China, and South Africa) and other G20 countries towards supporting health R&D globally [6].

If diseases long associated with poor countries, such as NTDs and other infections, are increasing among poor people in richer countries, what about diseases associated with rich countries? Noncommunicable diseases (NCDs), including such rich-country scourges as coronary artery disease, have for some years been increasing as causes of illness and death in low- and middle-income countries [7]. According to WHO’s *Global Status Report on NCDs 2014* for the year 2012, globally approximately 38 million people died from NCDs, from a total of 56 million people who died in that year. The WHO finds that four major disease groups—cancer, cardiovascular diseases, chronic respiratory diseases, and diabetes—are responsible for 82% of the NCD-related deaths. Among them, cardiovascular diseases accounted for almost one-half of the deaths (17.5 million), followed by cancers (8.2 million), respiratory diseases such as asthma and chronic obstructive pulmonary disease (4.0 million), and diabetes (1.5 million) [8].

Are increases in NCDs limited to growing higher-income classes? In 2013, the G20 nations and Nigeria accounted for almost 90% of the global economy, as estimated by total global gross domestic product (GDP). While these nations represent the world’s economic engine, the G20 nations and Nigeria also produce most of the world’s neglected infectious diseases (including NTDs) and most of its NCDs. Shown in Table 1 is the NCD mortality for the G20 countries and Nigeria, indicating that the world’s wealthiest economies account for approximately 26.5 million deaths from NCDs, or 70% of the world’s NCD-related deaths. The age-standardized death rates clearly indicate that, among the world’s leading economic powers, a nation’s GDP by itself is a poor indicator of its inhabitants’ risk of dying from NCDs.

**Table 1:** NCD mortality estimates in G20 countries + Nigeria, 2012^1^.

Country	GDP Rank^2^	Age-Standardized Death Rate per 100,000 from NCDs in Males	Age-Standardized Death Rate per 100,000 from NCDs in Females	Total Deaths from NCDs in the Year 2012
European Union	1			
United States	2	488.9	350.1	2.3 million
China	3	650.6	508.5	8.6 million
Japan	4	333.3	173.5	0.9 million
Germany	5	447.8	295.1	0.8 million
United Kingdom	6	425.9	302.2	0.5 million
France	7	412.7	234.8	0.5 million
Brazil	8	617.7	429.4	1.0 million
Italy	9	382.1	242.5	0.5 million
India	10	785.0	586.6	5.9 million
Russia	11	1,155.6	573.8	1.8 million
Canada	12	378.6	268.0	0.2 million
Australia	13	359.9	253.0	0.1 million
South Korea	15	415.0	221.4	0.2 million
Mexico	16	539.5	410.9	0.5 million
Indonesia	17	774.6	600.2	1.1 million
Turkey	19	726.3	430.8	0.4 million
Saudi Arabia	20	622.4	472.2	0.1 million
Nigeria	23	712.8	638.4	0.5 million
Argentina	25	599.4	370.9	0.3 million
South Africa	34	902.8	587.4	0.3 million
All G20 countries + Nigeria				26.5 million
Global				38 million
Percentage of global NCD deaths occurring in the G20 + Nigeria				70%

^1^ Data from reference [8]’s Table 4.2, “NCD mortality—Comparable estimates of NCD mortality (age-standardized death rates for NCDs per 100 000; total NCD deaths in 1000s ), 2012,” except for the 38 million global figure, which was obtained from the document text.

^2^ Rank from http://databank.worldbank.org/data/download/GDP.pdf, with the number one ranking for the European Union added.

These WHO data do not distinguish between wealthy and poor populations within the G20 nations and Nigeria. However, previous work indicates that at least some NCDs have been associated with lower, rather than higher, socioeconomic status within LMICs [9]. Moreover, NCDs are on the decline in high-income countries but rising among LMICs, where most of the world’s NCD-related deaths (especially premature deaths) now occur [8]. Taken together with these findings, the wide range of NCD mortality rates among G20 countries, with particularly high rates among non-high-income G20 countries, seems consistent with a hypothesis that the poor living among the wealthy disproportionately share the burden of NCDs globally. In other words, NCDs may be joining NTDs, HIV, TB, and malaria in following the familiar blue marble health pattern of geographical redistribution with convergence on the world’s poorest people.

These observations indicate the relevance of a blue marble health perspective in reshaping global health policy to include a spotlight on the G20 nations and Nigeria and targeting HIV/AIDS, tuberculosis, malaria, NTDs, and NCDs at future G20 summits. A specific component may include renewed commitments by these specific nations for access to health care and essential medicines for their own impoverished populations as well as those of lower-income countries. To this end, G20 nations should support the nine major targets that WHO has set to reduce global deaths from NCDs, with an overarching goal to reduce NCD deaths by one-quarter [8]. Important components include dietary and lifestyle changes, as well as access to essential medicines to control high blood sugar, high blood pressure, and other risk factors for cardiovascular diseases. In addition, there are a wide range of neglected causes of NCDs that are unique to people who live in extreme poverty [10] Ultimately, the world economic powers should also support R&D to address their own health disparities [11]. Recently, a global biomedical R&D fund has been proposed [5], which may be especially relevant to the G20 nations and Nigeria not only as supporters of improving health in less wealthy countries, but also as potential recipients of advances that will benefit the health of their own populations.

Such measures must not be enacted while failing to address poverty and other social determinants underlying the patterns that blue marble health aims to improve. As Michael Marmot and others have noted, “Social determinants are relevant to communicable and non-communicable disease alike. Health status, therefore, should be of concern to policy makers in every sector, not solely those involved in health policy” [12]. In parallel, global efforts should ensure that implementation of public health measures does not inadvertently widen health inequalities, as recently found for some cardiovascular disease prevention measures [13]. Strong economies must take responsibility for population-wide preventive action that embraces vulnerable populations now living in extreme poverty.

For research addressing the disparate burden of disease upon poor people across a variety of settings, *PLOS Medicine* and *PLOS Neglected Tropical Diseases* continue to encourage submissions to the recently updated Blue Marble Health Collection [1,14].

## Abbreviations:

G20: group of 20
GDP: gross domestic product
NCD: noncommunicable disease
NTD: neglected tropical disease
R&D: research and development
UNAIDS: Joint United Nations Programme on HIV/AIDS
WHO: World Health Organization
